# Intramolecular Azide to Alkene Cycloadditions for the Construction of Pyrrolobenzodiazepines and Azetidino-Benzodiazepines

**DOI:** 10.3390/molecules191016737

**Published:** 2014-10-17

**Authors:** Karl Hemming, Christopher S. Chambers, Faisal Jamshaid, Paul A. O’Gorman

**Affiliations:** 1 Department of Chemical Sciences, University of Huddersfield, Queensgate, Huddersfield, West Yorkshire HD1 3DH, UK; 2 Laboratory of Biotransformation, Institute of Microbiology, Academy of Sciences of Czech Republic, Vídeňská 1083, Praha 4, 142 20, Czech Republic

**Keywords:** cycloaddition, 1,3-dipole, azide, pyrrolobenzodiazepine, azetidinone, antitumour, antibiotic, β-lactam

## Abstract

The coupling of proline- and azetidinone-substituted alkenes to 2-azidobenzoic and 2-azidobenzenesulfonic acid gives precursors that undergo intramolecular azide to alkene 1,3-dipolar cycloadditions to give imine-, triazoline- or aziridine-containing pyrrolo[1,4]benzodiazepines (PBDs), pyrrolo[1,2,5]benzothiadiazepines (PBTDs), and azetidino[1,4]benzodiazepines. The imines and aziridines are formed after loss of nitrogen from a triazoline cycloadduct. The PBDs are a potent class of antitumour antibiotics.

## 1. Introduction

**Figure 1:**
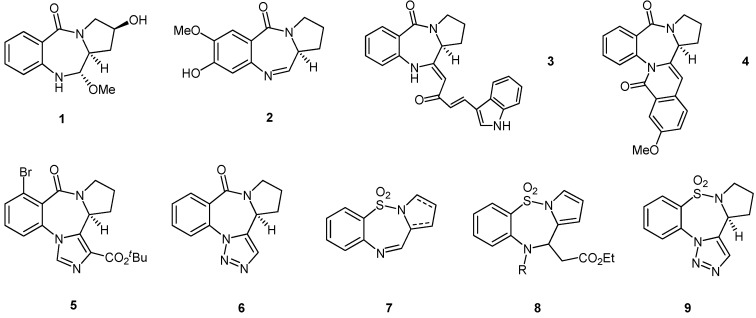
Pyrrolobenzodiazepine (PBD) and pyrrolobenzothiadiazepine (PBTD) structures of interest.

The pyrrolobenzodiazepines (PBDs), of which the natural products abbeymycin (**1**), DC-81 (**2**) and fuligocandin B (**3**) (Figure 1) are typical examples, are a class of molecule that has attracted significant interest due the antitumour and antibiotic activity of several members [1,2,3,4,5]. Synthetic hybrids [5,6,7,8] and dimers [5,9,10,11] have also shown significant biological activity and members of the dimer class have entered Phase II clinical development as antitumour compounds. PBDs with additional fused rings such as the circumdatin (**4**) [12], bretazenil (**5**) [13] and the 1,2,3-triazolo-fused system **6** [14] are attractive as potential antitumour compounds, neurological agents, and protease inhibitors, respectively. As a result of this interest, the replacement of the PBD pyrrole ring with other rings has attracted attention [5,15] and hence pyrido- [16], oxazolo- [17], thiazolo- [18] and imidazo- [19,20,21] analogues are all known. The replacement of the PBD tertiary amide with a sulfonamide leads to the pyrrolobenzothiadiazepines (PBTDs) which are synthetic analogues of the PBDs [22]. The PBTDs have received much less attention than the PBDs, but it is known that PBTDs **7**–**9** are useful as non-nucleosidic reverse transcriptase inhibitors [23], potent leukemia cell-line inhibitors [24] and Glut-1 transporter inhibitors with chemotherapeutic potential [25], respectively.

**Figure 2:**
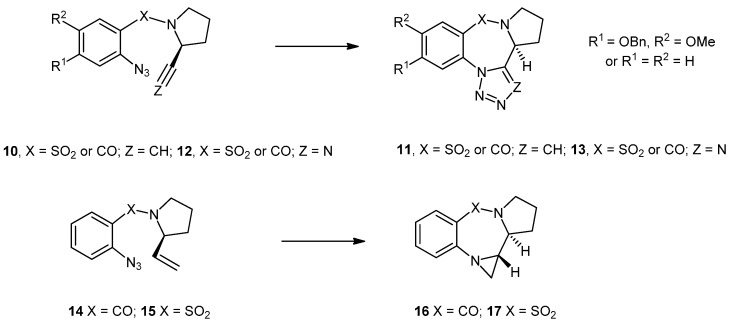
Previous examples of PBDs and PBTDs synthesized by 1,3-dipolar cycloaddition.

We reported previously [26] that the 1,2,3-triazolo-fused PBDs and PBTDs **11** (Figure 2) are available via intramolecular azide to alkyne 1,3-dipolar cycloaddition of 1-(azidoaryl)-2-alkynyl pyrroles **10**, and have recently shown [25] that the 1-(azidoaryl)-2-cyano pyrroles **12** give the 1,2,3,4-tetrazolo-fused PBD and PBTD systems **13**. 

**Figure 3:**
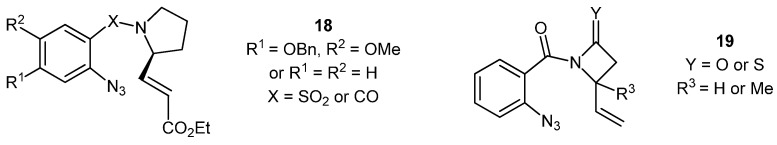
Additional alkene based substrates studied in this paper.

In preliminary work involving alkenes, we demonstrated that 1-(azidoaryl)-2-alkenyl pyrroles **14** and **15** gave the aziridino-fused PBD **16** and PBTD **17** [27]. In this paper, we report the full details for the synthesis of compounds **16** and **17** and also discuss several new intramolecular cycloadditions involving the previously unreported 1-(azidoaryl)-2-alkenyl pyrroles **18** (Figure 3) along with the reactions of a second new series of compounds, the 1-(azidoaryl)-4-alkenyl-azetidin-2-ones **19**.

## 2. Results and Discussion

### 2.1. Synthesis and Reactivity of the Pyrrolo-Based Systems 

**Scheme 1:**
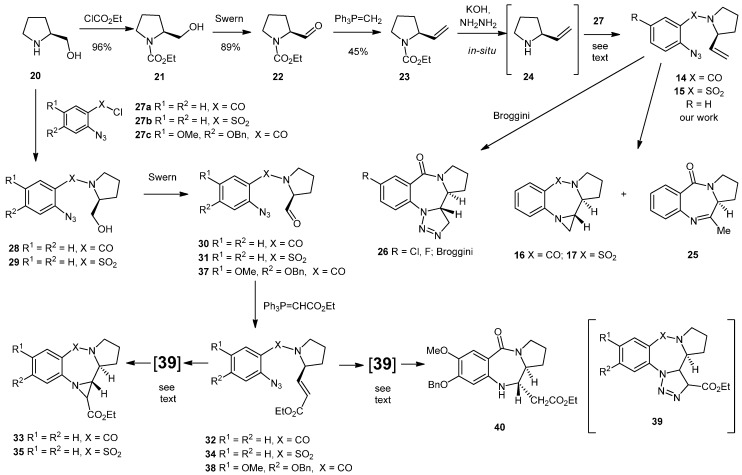
Synthesis of aziridinopyrrolobenzodiazepines and aziridinopyrrolobenzothiadiazepines.

The largest and most researched class of PBDs, those represented by abbeymycin (**1**) and DC-81 (**2**), are able to interact with nucleophilic guanidine residues in the minor groove of DNA, a process that is reliant upon the presence of an electrophilic imine or carbinolamine based functional group [5]. It is of note that the (*S*)-chiral center that is present in the 3-dimensional structure of these PBDs gives the molecules a shape which enables them to twist into the DNA minor groove meaning that most syntheses are based upon derivatives of (*S*)-proline. It is known that intramolecular 1,3-dipolar cycloadditions of azides to alkenes can lead to either imines or aziridines via triazoline intermediates [28,29]. On this basis, we anticipated that we could access imine or aziridine containing PBDs using intramolecular 1,3-dipolar cycloadditions between azides and alkenes using alkenes derived from (*S*)-proline. Whilst we were interested in the possibility of producing an imine, we were more intrigued by the possibility of producing an aziridine due the known propensity [30] of aziridines to function as electrophiles and the potential biological activity that a novel system of this type might offer. We settled upon two approaches, as shown in Scheme 1.

In the first system, (*S*)-prolinol (**20**) was converted into the unstable alkene **24** using a modified literature protocol [31]. Thus, *S*-prolinol (**20**) was protected as the carbamate **21**, oxidized to the aldehyde **22** and then converted to the N-protected alkene **23** by Wittig reaction. *In-situ* deprotection and coupling of the deprotected alkene **24** to 2-azidobenzoyl chloride or 2-azidobenzenesulfonyl chloride **27a/b** gave the cycloaddition precursors **14** and **15** in 32% and 20% yield, respectively, from the carbamate **23**. Heating compound **14** in chloroform gave a ~1:1 mixture of the imine **25** and the aziridine **16** (55% combined yield) whereas heating compound **15** in the same solvent gave the aziridine **17** as the only isolable product in 44% yield.

We assume these reactions proceed via intramolecular 1,3-dipolar cycloaddition to give an intermediate triazoline which then undergoes loss of two nitrogen atoms. It is of note that Broggini explored a similar process with halogenated aryl systems and obtained the triazolines **26** [32]. The presence of the aziridine ring in compounds **16** and **17** was clear in the NMR spectra from the extra CH_2_ and CH groups and from the diagnostic lack of CH_2_ geminal coupling in the aziridine CH_2_.

Mass spectroscopy confirmed loss of two nitrogen atoms and precluded the triazoline. The relative stereochemical assignment in compounds **16** and **17** was made on the basis of the CH/CH coupling constant at 8–10 Hz, the lack of a CH to CH nOesy correlation and the presence of aziridine CH_2_ to pyrrolidine CH correlation, and the unequivocal assignment of Broggini's system by X-ray crystallography [32]. Stereochemistry at the aziridine nitrogen was not determined. Although we used (*S*)-prolinol as the starting material (due to its ease of availability), we did not seek to confirm absolute stereochemical assignments, although it is of note that Sato and co-workers reported that the alkene **24** retains the (*S*)-configured centre [31].

In our second approach, also shown in Scheme 1, we coupled (*S*)-prolinol to 2-azidobenzoyl chloride or 2-azidobenzenesulfonyl chloride **27a/b** (80%–96%) and oxidized the alcohols **28** and **29** under Swern conditions to give the aldehydes **30** and **31** (72%–80%). Attempts to react the aldehydes **30** and **31** with (methylene)triphenylphosphorane were unsuccessful meaning that a more efficient route (compared to the synthesis and *in-situ* deprotection of **23**) to the alkenes **14** and **15** was not possible. However, reactions of these aldehydes with (carbethoxymethylene)triphenylphosphorane in toluene were successful. In the case of reaction with aldehyde **30**, the alkene **32** was isolated in 51% yield and then heated in chloroform, whereupon it converted into the aziridine **33** as a 1:1 mixture of diastereoisomers in 30% yield. In the case of aldehyde **31**, the alkene **34** could not be isolated and the aziridine **35** was isolated as the only product in 34% yield from the aldehyde, this time as a single diastereoisomer, possibly due to the lower temperature at which reaction occurred. We assigned the aziridine-CH/pyrrolidine-CH stereochemistry on the same basis as that described above, but were unable to determine the stereochemistry at the CHCO_2_Et chiral center. We assume that these products arise as a result of azide to alkene cycloaddition and formation of an intermediate triazoline **39**, which then undergoes loss of two nitrogen atoms. As discussed previously, this loss of nitrogen is known behavior in alkene to azide cycloaddition processes [28,29]. We also studied reactions involving (2-azido-4-benzyloxy-5-methoxy)benzoyl chloride **27c** (X = CO, R^1^ = OMe, R^2^ = OBn) due to this having the substitution pattern present in the natural product DC-81 (**2**). This azide was available in six steps from commercially available 4-hydroxy-3-methoxybenzoic acid using a literature procedure [33,34]. Attempts to couple this acid chloride to the alkene **24** were unsuccessful and led to significant degradation of the azide staring material–possibly as a result of its intolerance to the extreme *in-situ* conditions under which the alkene **24** was generated [31]. However, coupling of the acid chloride **27c** to prolinol was successful and subsequent oxidation gave the aldehyde **37**. Attempted reaction of this aldehyde with (methylene)triphenylphosphorane was again unsuccessful. However, reaction with (carbethoxymethylene)triphenylphosphorane gave a new product. As was observed previously with aldehyde **31**, the alkene **38** was not isolable. This time, however, the product was unexpectedly found to be the “reduced” pyrrolobenzodiazepine **40**, formed as a single diastereoisomer in 21% yield from the aldehyde, with stereochemistry consistent with that discussed above (no CH to CH correlation by nOesy and a strong CH_2_CO_2_Et to pyrrolidine CH correlation). We assume that this product arises as a result of a free-radical based loss of nitrogen from the triazoline **39** followed by hydrogen abstraction from the toluene solvent rather than imine/aziridine formation as observed previously.

### 2.2. Synthesis and Reactivity of the Azetidino Based Systems

**Scheme 2:**
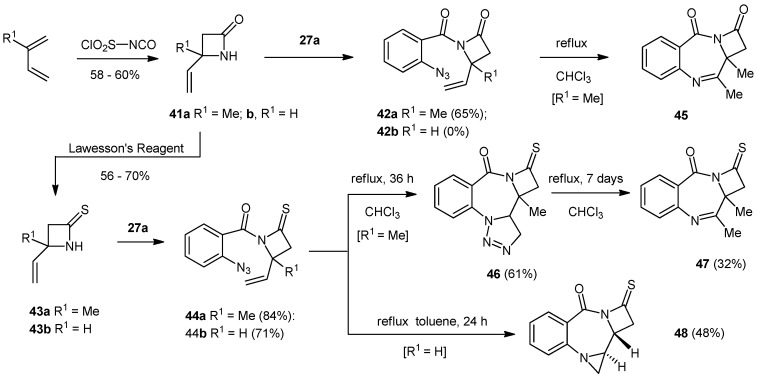
Synthesis and reactivity of 1-(2'-azidobenzoyl)-4-alkenyl-1-azetidin-2-ones/2-thiones.

In order to obtain further examples of these cycloaddition processes, we next turned our attention to the use of 4-alkenyl-2-azetidinones (Scheme 2) as coupling partners for 2-azidobenzoyl chloride (**27a**). We chose azetidinone systems due to our long-standing interest in the chemistry and biological applications of the β-lactams and their derivatives [35,36], and also due to the lack of literature examples of azetidino analogues of the PBDs. The 4-alkenyl-2-azetidinones **41** were synthesized in 58%–60% yield using a [2+2]-cycloaddition between a diene and chlorosulfonyl isocyanate followed by work-up to remove the N-sulfonyl group. Whilst the 4-methyl substituted β-lactam **41a** coupled successfully (65% yield) to give the 1-(2'-azidobenzoyl)-2-azetidinone **42a**, we were unable to obtain the corresponding demethyl system **42b** from the β-lactam **41b**. We thus converted the azetidinones **41a/b** into the corresponding azetidinethiones **43a/b** by reaction with Lawesson's reagent, reasoning that this may result in a nitrogen atom that was more nucleophilic, and in fact, the two azetidin-2-thiones **43a/b** reacted successfully with 2-azidobenzoyl chloride (**27a**) to give the 1-(2'-azidobenzoyl)-2-azetidinthiones **44a/b** in 84% and 71% yield, respectively. Each of the three azido alkenes **42a** and **44a/b** behaved differently when heated to reflux in boiling solvents. Compound **42a** gave the imine **45** (38%) when heated in boiling chloroform for 72 h; compound **44a** when heated in boiling chloroform also gave the corresponding imine **47**, but this time formed the isolable triazoline **46** (61% yield) after 72 h and gave the imine in 32% yield after a further 7 days in boiling chloroform; compound **44b** was stable in chloroform at reflux, but gave the aziridine **48** (48% yield) upon 24 h of heating in toluene at reflux. The aziridine ring in compound **48** was apparent from the CH_2_CHCHCH_2_ connectivity in the system, the distinctive lack of geminal proton coupling for the aziridine CH_2_ and the loss of two nitrogen atoms in the mass spectrum. The product was formed as a single diastereoisomer according to the ^1^H/^13^C-NMR spectra. Stereochemistry at the aziridine nitrogen was not determined. The CH/CH stereochemistry was assigned by correlation to the systems described above on the basis of the similar 8.9 Hz coupling constant.

## 3. Experimental Section

### 3.1. General Information

Reactions were performed in oven-dried glassware under nitrogen dried through 4 Å molecular sieves and delivered through a gas manifold. Work-up procedures were carried out in air. Anhydrous grade solvents were freshly distilled using a continuous still under nitrogen. Chloroform was dried over 4 Å molecular sieves or distilled over phosphorus pentoxide (3% w/v). Dichloromethane, acetonitrile, ethyl acetate and toluene were distilled over calcium hydride (5% w/v) over 4–6 h. Diethyl ether and THF were pre-dried over sodium wire, and then distilled over sodium wire (1%–2% w/v) with benzophenone (0.2%–0.3% w/v) as an indicator. Other anhydrous solvents were purchased from Fisher Chemicals or Sigma-Aldrich. All reactions were monitored by TLC, which was carried out on 0.20 mm Macherey-Nagel Alugram^®^ Sil G/UV_254_ silica gel-60 F_254_ precoated aluminium plates and visualisation was achieved using UV light or vanillin stain. Column chromatography was performed on Merck silica gel (0.063–0.200 mm, 60 Å). NMR spectra were recorded on a Bruker DPX-400 or on a Bruker Avance 500 instrument. IR spectra were recorded on a Nicolet 380 FT-IR instrument as a drop for oils and liquids or as neat powders for solids. Mass spectra were recorded on a Bruker Daltonics micrOTOF mass spectrometer operating at a positive ion mode under an electrospray ionization (ESI +) method. High resolution mass spectra were recorded on a Finnegan MAT 900 XTL instrument operated by the EPSRC National Mass Spectrometry service at the University of Swansea. 1-(2'-Azidobenzoyl)pyrrolidine-2-carbaldehyde [33,34], 2-azidobenzenesulfonic acid [37] and 2'-azido-4-(benzyloxy)-5-methoxybenzoic acid [33,34] were synthesized as described in the literature. The synthesis of *N*-2-ethenyl-1-ethoxycarbonylpyrrolidine (**23**) and its deprotection have been described previously [31], but full details are included below as we found some modification was necessary. Due to ease of availability, we started our synthesis with (*S*)-prolinol, but we did not seek to confirm the absolute stereochemical integrity of subsequent materials. Full experimental procedures for the synthesis of compounds **16**, **17** and **25** have not appeared previously, although we have reported this chemistry in preliminary form as part of an earlier study [27]. Similarly, we have described the synthesis and use of compounds **41** and **43** before as precursors in other chemistry [36], but have not before given detailed experimental procedures. Compounds **32**–**35**, **38**, **40**, **42** and **44**–**48** are previously unreported.

### 3.2. Synthesis and Reactivity of the Pyrrolo-Based Systems

*N-(Ethoxycarbonyl)-prolinol* (**21**). To a stirring solution of *S*-prolinol (**20**, 1.00 g, 9.89 mmol) in 4 M NaOH (7 mL) was added ethyl chloroformate (1.13 mL, 1.29 g, 11.9 mmol) over 10 min at 0 °C. The reaction was stirred at 0 °C for 30 min followed by 30 min at ambient temperature. The reaction solution was neutralized with 2M HCl, the aqueous phase was separated and extracted with DCM (3 × 10 mL). The combined organic layers were dried (MgSO_4_), filtered and concentrated under reduced pressure to give the crude product **21** (1.64 g, 96%) as a yellow oil which was used directly in the next step. NMR: δ_H_ (400 MHz, CDCl_3_): 1.27 (3H, t, *J* = 7.1, COCH_2_C*H*_3_), 1.57 (1H, m, C*H*H), 1.74–1.91 (2H, m, CH*H* + C*H*H), 1.99–2.08 (1H, m, CH*H*), 3.31–3.78 (1H, m, NC*H*H), 3.49–3.54 (1H, m, NCH*H*), 3.59–3.69 (2H, m, CH_2_OH), 3.97–4.03 (1H, m, CH_2_O*H*), 4.14 (2H, q, *J* = 7.1, COCH_2_CH_3_), 4.61 (1H, dd, *J* = 7.6, 2.6, NC*H*CH_2_); δ_C_ (100 MHz, CDCl_3_): 14.7 (CH_3_), 24.1 (CH_2_), 28.6 (CH_2_), 47.3 (CH_2_), 60.6 (CH), 61.6 (CH_2_), 67.3 (CH_2_), 157.6 (q). IR: υ_max_ (thin film cm^−1^): 770 (s), 906 (m), 1046 (s), 1106 (s), 1333 (s), 1379 (s), 1414 (s), 1667 (s), 2876 (w), 2975 (w), 3400–3500 (br). LRMS (ESI+): Found 196.1 [M+Na]^+^, C_8_H_15_N_4_NaO_3_ requires 196.1.

*N-(Ethoxycarbonyl)-prolinal* (**22**). A 2 M solution of (COCl)_2_ in DCM (4.72 mL, 9.43 mmol) was diluted with dry DCM (12 mL) and cooled to −78 °C under N_2_. DMSO (1.34 mL, 1.47 g, 18.9 mmol) in DCM (5 mL) was added followed by the alcohol **21** (1.36 g, 7.86 mmol) in DCM (5 mL), over 15 min. The whole was maintained at −78 °C for 30 min before the addition of Et_3_N (5.48 mL, 3.98 g, 39.3 mmol) dropwise over 10 min. The whole was allowed to reach room temperature over an hour before being quenched with a mixture of Et_2_O (12.5 mL) and H_2_O (12.5 mL). The organic layer was separated and the aqueous phase was extracted with DCM (3 × 10 mL). The combined organics were dried (MgSO_4_), filtered, concentrated under reduced pressure and purified by silica chromatography (20 g) (EtOAc/Hex; 2:3) to yield the aldehyde **22** as a mixture of rotamers in the form of a yellow oil (1.19 g, 89%). NMR: δ_H_ (400 MHz, CDCl_3_): 1.17 & 1.24 (3H, 2 × t, *J* = 7.1 & 7.1, COCH_2_C*H*_3_), 1.76–1.92 (2H, m, CH_2_), 1.93–2.09 (2H, m, CH_2_), 3.40–3.57 (2H, m, NCH_2_), 4.02–4.14 & 4.17–4.22 (3H, m, CO_2_CH_2_CH_3_ & NC*H*CHO), 9.48 & 9.56 (1H, 2 × d, *J* = 2.5, 1.7, CHO); δ_C_ (100 MHz, CDCl_3_): 14.5/14.7 (CH_3_), 23.8/24.5 (CH_2_), 26.6/27.8 (CH_2_), 46.6/47.1 (CH_2_), 61.5 (2 × CH_2_), 64.8/65.1 (CH), 154.7/155.6 (q), 200.2/200.3 (CHO). IR: υ_max_ (thin film cm^−1^): 729 (s), 771 (s), 914 (m), 1021 (m), 1102 (s), 1172 (m), 1341 (s), 1380 (s), 1416 (s), 1466 (s), 1687 (s), 1733 (s), 2872 (m), 2980 (m). LRMS (ESI+): Found 194.1 [M+Na]^+^, C_8_H_13_NNaO_3_ requires 194.1.

*N-2-Ethenyl-1-ethoxycarbonylpyrrolidine* (**23**)*.* *n*-BuLi in hexanes (1.6 M, 10.5 mL, 16.8 mmol) was added dropwise over 30 min to a stirring suspension of methyltriphenylphosphonium bromide (5.52 g, 15.4 mmol) in anhydrous THF (30 mL) at −78 °C under an inert atmosphere of nitrogen. The whole was allowed to reach −10 °C and kept at that temperature for 30 min before being cooled back to −78 °C. The aldehyde **22** (1.20 g, 7.02 mmol) in anhydrous THF (5 mL) was added dropwise over 10 min. The whole was allowed to warm to −10 °C, and maintained at that temperature for 2 h and then the mixture was allowed to reach room temperature overnight. The mixture was quenched with saturated aqueous NH_4_Cl (20 mL) and the aqueous layer was separated and extracted with EtOAc (3 × 10 mL). The combined organic layers were dried (MgSO_4_), filtered, concentrated under reduced pressure and purified by silica chromatography (50 g) (EtOAc/Hex; 3:2) yielding an air sensitive product as a mixture of rotamers in the form of a pale orange oil (536 mg, 45%). NMR: δ_H_ (400 MHz, CDCl_3_): 1.22–1.27 (3H, br, m, CH_3_), 1.71 (1H, bs, CH_2_CH*H*CH_2_), 1.81 (2H, br, m, NCHCH_2_), 1.97 (1H, m, br, CH_2_C*H*HCH_2_), 3.43 (2H, s, br, NCH_2_), 4.13 (2H, br, m, OCH_2_), 4.33 (1H, br, m, NCH), 5.04–5.12 (2H, br, m, CH=C*H*_2_), 5.72 (1H, br, m, C*H*=CH_2_); δ_C_ (100 MHz, CDCl_3_): 14.2/14.8 (CH_3_), 22.6/23.4 (CH_2_), 31.2/31.9 (CH_2_), 46.3/46.5 (CH_2_), 58.9/59.3 (CH), 60.4/60.8 (CH_2_), 113.8/114.1 (CH_2_), 138.2/138.5 (CH), 155.1/155.4 (q).

*N-(2'-Azidobenzoyl)-2-ethenylpyrrolidine* (**14**). To a vigorously stirred suspension of finely ground KOH (2.31 g, 41.2 mmol) in ethylene glycol (7.2 mL) was added hydrazine hydrate (0.25 mL, 7.92 mmol) and 2-ethenyl-1-ethoxycarbonylpyrrolidine (**23**) (0.2706 g, 1.58 mmol) and the whole was heated to reflux (195 °C) for 4 h. The reaction was allowed to reach ambient temperature before being diluted with a mixture of Et_2_O (4 mL) and H_2_O (4 mL). The thick syrup was extracted with Et_2_O (3 × 5 mL) and dried with finely ground NaOH. Et_3_N (0.33 mL, 2.37 mmol) was added to the ethereal solution containing the amine at 0 °C under an inert atmosphere and was stirred for 10 min before the freshly prepared acid chloride **27a** (0.48 g, 2.30 mmol) in Et_2_O (5 mL) was added dropwise over 10 min and the whole was allowed to reach ambient temperature overnight. The reaction was diluted with water (20 mL), the ethereal layer was separated and the aqueous phase was extracted with ether (3 × 10 mL). The combined organics were dried (MgSO_4_), filtered, concentrated under reduced pressure and purified by silica chromatography (21 g) (EtOAc/Hex; 2:3) to yield the product as a mixture of rotamers (0.1230 g, 32%) in the form of a yellow oil. NMR: δ_H_ (400 MHz, CDCl_3_): 1.73–2.15 (4H, m, 2 × CH_2_), 3.17–3.23 & 3.30–3.36 (1H, m, C*H*HCH_2_), 3.64–3.71 & 3.74–3.82 (1H, m, CH*H*CH_2_), 4.11–4.15 & 4.84–4.87 (1H, br, m, C*H*CH_2_), 4.70 & 5.35 (1H, 2 × d, *J* = 16.9, 17.1, C*H*HCH), 4.89 & 5.20 (1H, 2 × d, *J* = 10.3 & 10.4, CHCH*H*), 4.89 & 5.20 (1H, 2 × d, *J* = 10.3 & 10.4, CHC*H*H), 5.56 & 5.90 (1H, 2 × ddd, *J* = 6.2, 10.4, 16.9 & 4.8, 10.4, 17.1, C*H*CH_2_) 7.10–7.24 (3H, m, ArH), 7.32–7.45 (1H, m, ArH); δ_C_ (100 MHz, CDCl_3_): 22.1/23.6 (CH_2_), 30.9/32.3 (CH_2_), 45.9/48.2 (CH_2_), 58.4/61.1 (CH), 114.5/114.9 (CH_2_), 118.4/118.5 (CH), 124.6/125.2 (CH), 127.9/128.3 (CH), 129.6/130.1 (q), 130.2/130.3 (CH), 136.1 (q), 136.9/137.6 (CH), 166.8/167.4 (q). IR: υ_max_ (thin film cm^−1^): 750 (s), 932 (s), 1083 (m), 1149 (m), 1292 (s), 1415 (s), 1449 (s), 1479 (s), 1598 (s), 1631 (s), 2128 (s), 2878 (m), 2973 (m), 3078 (w). LRMS (ESI+): Found 265.1 [M+Na]^+^, 507.2 [2M+Na]^+^. HRMS (ESI+): Found 265.1064 [M+Na]^+^, C_13_H_14_N_4_NaO requires 265.1060.

*Pyrrolobenzodiazepine* (**25**) *and aziridinopyrrolobenzodiazepine* (**16**). The alkene **14** (78.0 mg, 0.32 mmol) was dissolved in CHCl_3_ (10 mL) and heated at reflux under an inert atmosphere of nitrogen for 16 h whilst being monitored by TLC (EtOAc/Hex; 3:2). The reaction mixture was concentrated under reduced pressure and purified by silica chromatography (20 g) (EtOAc/Hex; 3:2–4:1) to yield two inseparable close-running spots on TLC which were found to be the aziridine **16** and the methyl imine **25** in a 1:1 ratio (estimated by ^1^H-NMR, 43 mg, 55%). NMR: δ_H_ (400 MHz, CDCl_3_): 1.96–2.06 (2H, m, CH_2_), 2.00 (1H, d, *J* = 3.6, aziridine CH_2_), 2.09–2.22 (2H, m, 2 × C*H*H), 2.18–2.26 (3H, m, CH_2_ + C*H*H), 2.27 (3H, s, CH_3_), 2.29–2.37 (1H, m, C*H*H), 2.53 (1H, d, *J* = 4.3, aziridine CH_2_), 2.78 (1H, ddd, *J* = 9.5, 4.3, 3.6, aziridine CH), 3.34 (1H, ddd, *J =* 9.5, 2.9, 1.6, NCH [aziridine]), 3.45–3.52 (1H, m, NCH), 3.56–3.63 (1H, m, NCH*H*), 3.62–3.72 (2H, m, 2 × NCH), 3.74–3.84 (1H, m, NCH_2_), 3.81–3.95 (1H, m, NC*H_2_*), 7.01 (1H, dt, *J* 7.9, 0.7, Ar*H*), 7.11 (1H, d, *J* 8.1, Ar*H*), 7.14–7.18 (2H, m, 2 × ArH), 7.40 (1H, dt, *J* = 7.6, 1.6, ArH), 7.44–7.52 (1H, m, Ar*H*), 7.74 (1H, d, *J* = 7.9, Ar*H*), 7.93 (1H, dd, *J* = 7.9, 1.6, ArH); δ_C_ (100 MHz, CDCl_3_): 22.4 (CH_3_), 23.1 (*C*H_2_), 24.2 (CH_2_), 27.9 (CH_2_), 29.4 (*C*H_2_), 32.7 (*C*H_2_), 32.9 (CH_2_), 44.8 (*C*H), 46.1 (*C*H_2_), 55.6 (CH), 58.1 (*C*H), 122.0 (*C*H), 122.9 (*C*H), 125.5 (CH), 126.4 (CH), 126.8 (q),127.0 (q), 129.7 (*C*H),129.8 (CH), 131.2 (*C*H), 131.4 (CH), 145.6 (q), 145.7 (q), 150.3 (q), 165.5 (q), 169.9 (q). IR: υ_max_ (thin film cm^−1^): 907 (s), 1244 (m), 1320 (m), 1403 (m), 1454 (s), 1614 (s), 1681 (m), 1743 (m), 2876 (w), 2924 (m), 2970 (w). LRMS (ESI+): Found 215.1 [M+H]^+^, 237.1 [M+Na]^+^, 451.2 [2M+Na]^+^, 665.3 [3M+Na]^+^. HRMS (ESI+): Found 215.1179 [M+H]^+^, C_13_H_15_N_2_O requires 215.1179.

*N-(2'-Azidobenzenesulfonyl)-2-ethenylpyrrolidine* (**15**). To a stirring suspension of KOH (4.280 g, 76.5 mmol) in ethylene glycol (15 mL), hydrazine hydrate (0.46 mL, 471 mg, 14.7 mmol) was added under nitrogen followed by the ester protected alkene **23** (497 mg, 2.94 mmol), and the whole was heated at reflux (195 °C) for 4.5 h. The reaction mixture was cooled to ambient temperature and was diluted with Et_2_O (9 mL) and water H_2_O (9 mL). The organic layer was separated and the aqueous layer was extracted with Et_2_O (3 × 5 mL) and dried over NaOH. Et_3_N (0.62 mL, 4.41 mmol) was added to the ethereal solution containing the amine at 0 °C and the mixture was stirred for 10 min under an inert atmosphere of nitrogen before addition of freshly prepared 2-azidobenzenesulfonyl chloride **27b** [which was prepared by heating at reflux 2-azidobenzenesulfonic acid (900 mg, 4.41 mmol) with 2M oxalyl chloride in DCM (4.4 mL, 8.82 mmol) which was concentrated under reduced pressure and suspended in Et_2_O (10 mL)]. The whole was allowed to reach room temperature overnight. The reaction was diluted with water (30 mL), the ethereal layer was separated and the aqueous layer was extracted with Et_2_O (3 × 10 mL). The combined organics were dried (MgSO_4_), filtered, concentrated under reduced pressure and purified by silica chromatography (20 g) (EtOAc/Hex; 1:4) to yield the product as a yellow oil (163 mg, 20%). NMR: δ_H_ (400 MHz, CDCl_3_): 1.30–1.40 (1H, m, C*H*H), 1.83–1.92 (2H, m, CH_2_), 2.07–2.10 (1H, m, CH*H*), 2.92–2.99 (1H, m, C*H*H), 3.54–3.57 (1H, m, CH*H*), 3.93–3.98 (1H, m, NCH), 4.37–4.40 (1H, m, CH=C*H*H), 4.54 (1H, dd, *J* = 12.6, 17.8, CH=CH*H*), 5.30–5.37 (1H, m, C*H*=CH_2_), 7.16 (1H, dd, *J* = 8.0, 8.0, ArH), 7.45 (1H, dd, *J* = 8.4, 8.4, ArH), 7.83 (1H, d, *J* = 8.4, ArH), 7.91 (1H, d, *J* = 8.0, ArH). IR: υ_max_ (thin film cm^−1^): 926 (m), 1026 (s), 1099 (m), 1157 (m), 1182 (s), 1296 (m), 1330 (m), 1445 (m), 1471 (s), 1518 (m), 1592 (s), 2137 (s), 2871 (m), 2977 (m). LRMS (ESI+): Found 301.1 [M+Na]^+^. HRMS (ESI+): Found 301.0722 [M+Na]^+^, C_12_H_14_N_4_O_2_S + Na^+^ requires 301.0730.

*Aziridinopyrrolobenzothiadiazepine* (**17**). The *N*-(2'-azidobenzenesulfonyl)-2-ethenylpyrrolidine (**15**) (125 mg, 0.449 mmol) was dissolved in toluene (10 mL) and heated to reflux for 18 h. The reaction was allowed to reach room temperature before the solvent was removed and the crude was purified by silica chromatography (25 g) (EtOAc/Hex;2:3) to yield the aziridine product **17** as a tan colored oil (50 mg, 44%). NMR: δ_H_ (500 MHz, CDCl_3_): 1.81–2.07 (4H, m, 2 × CH_2_), 2.91–2.95 (1H, m, SO_2_NC*H*H), 3.32 (1H, dd, *J* = 9.9, 11.7, ArNCH*H*), 3.50 (1H, m, SO_2_NCH*H*), 3.57 (1H, dd, *J =* 3.9, 11.7, ArNC*H*H), 3.89 (1H, ddd, *J =* 2.9, 2.9, 7.9, SO_2_NC*H*), 4.84–4.87 (1H, m, ArNC*H*), 6.70 (1H, d, *J =* 8.1, ArH), 6.84 (1H, dd, *J =* 7.8, 7.8, ArH), 7.20 (1H, ddd, *J =* 7.8, 7.8, 1.3, ArH), 7.70 (1H, dd, *J =* 8.1, 1.3, ArH); δ_C_ (125 MHz, CDCl_3_): 24.5 (CH_2_), 25.8 (CH_2_), 43.4 (CH_2_), 49.2 (CH_2_), 59.3 (CH), 62.9 (CH), 118.6 (CH), 119.9 (CH), 125.7 (q), 129.2 (CH), 132.7 (CH), 144.6 (q). IR: υ_max_ (thin film cm^−1^): 759 (s), 1009 (m), 1037 (m), 1081 (s), 1127 (s), 1274 (m), 1333 (s), 1482 (s), 1520 (m), 1591 (s), 2926 (m), 2970 (w), 3066 (m), 3362 (w). LRMS (ESI+): Found 273.1 [M+Na]^+^, 523.1 [2M+Na]^+^, C_12_H_14_N_2_NaO_2_S requires 273.1. HRMS (ESI+): Found 251.0845 [M+H]^+^, C_12_H_14_N_2_O_2_S + H^+^ requires 251.0849.

*Synthesis of 1-[2'-Azido-4-(benzyloxy)-5-methoxybenzoyl]prolinal* (**37**)

Step 1: Synthesis of 1-[2'-Azido-4-(benzyloxy)-5-methoxybenzoyl]prolinol

2'-Azido-4-(benzyloxy)-5-methoxybenzoic acid (236 mg, 0.789 mmol) was dissolved in dry toluene (5 mL) and SOCl_2_ (1.5 mL) was added. The solution was placed in a preheated oil bath at 85 °C for 3 h. The reaction was cooled to ambient temperature, concentrated *in vacuo*, dissolved in fresh DCM (3 × 10 mL) and concentrated *in vacuo*, to remove the excess SOCl_2_. K_2_CO_3_ (218 mg, 1.58 mmol) in water (2 mL) was added in one portion to a stirring solution of *S*-prolinol (0.162 mL, 168 mg, 1.66 mmol) in DCM (2 mL) and the whole was stirred for 10 min. The acid chloride in DCM (5 mL) was added drop-wise and the whole was stirred at ambient temperature overnight before the organic layer was separated. The aqueous phase was extracted with DCM (3 × 10 mL) and the combined organic layers were dried (MgSO_4_), filtered, concentrated and purified by silica chromatography (20 mg) (EtOAc/Hex; 3:1) to yield the product as an orange oil (262 mg, 87%). NMR: δ_H_ (400 MHz, CDCl_3_): 1.56–1.84 (3H, m, CH_2_ + C*H*H), 2.06–2.13 (1H, m, CH*H*), 3.20–3.30 (2H, m, CH_2_), 3.62–3.67 (1H, m, C*H*HOH), 3.73–3.77 (1H, m, CH*H*OH), 3.80 (3H, s, OMe), 4.23–4.29 (1H, m, C*H*CH_2_OH), 4.69 (1H, bs, CH_2_O*H*), 5.10 (2H, s, OCH_2_), 6.59 (1H, s, ArH), 6.76 (1H, s, ArH), 7.24–7.38 (5H, m, ArH); δ_C_ (100 MHz, CDCl_3_): 24.5 (CH_2_), 28.5 (CH_2_), 49.6 (CH_2_), 56.3 (CH_3_), 61.2 (CH), 66.6 (CH_2_), 71.2 (CH_2_), 104.2 (CH), 110.7 (CH), 121.4 (q), 127.3 (CH), 128.2 (CH), 128.7 (CH), 135.9 (q), 147.2 (q), 149.8 (q), 168.8 (q). IR: υ_max_ (thin film cm^−1^): 742 (s), 1004 (m), 1048 (m), 1077 (m), 1178 (s), 1214 (s), 1242 (s), 1433 (s), 1511 (s), 1603 (s), 2109 (s), 2882 (w), 2937 (w), 3145–3593 (br). LRMS (ESI+): Found 383.2 [M+H]^+^, 765.3 [2M+H]^+^, 787.3 [2M+Na]^+^. HRMS (ESI+): Found 383.1708 [M+H]^+^, C_20_H_22_N_4_O_4_ + H^+^ requires 383.1714.

Step 2: Oxidation to 1-[2'-azido-4-(benzyloxy)-5-methoxybenzoyl]prolinal (**37**)

DMSO (0.14 mL, 154 mg, 1.97 mmol) in DCM (1 mL) and the alcohol from the previous step (250 mg, 0.654 mmol) in DCM (2 mL) were added dropwise, respectively, over 15 min, to a solution of 2 M oxalyl chloride (0.39 mL, 0.785 mmol) at −78 °C in DCM (1 mL). After 10 min, Et_3_N (0.24 mL, 174 mg, 1.72 mmol) was added dropwise to the mixture at −78 °C. The whole was allowed to reach room temperature over two h before being quenched with a mixture of Et_2_O (10 mL) and H_2_O (10 mL). The organic layer was separated and the aqueous layer was extracted with DCM (3 × 10 mL). The combined organics were dried (MgSO_4_), filtered, concentrated and purified by silica chromatography (20 g) (EtOAc/Hex; 3:1) to give the product as a mixture of rotamers in the form of an orange oil (161 mg, 65%). NMR: δ_H_ (400 MHz, CDCl_3_): 1.79–1.89 (2H, m, CH_2_), 1.97–2.04 (1H, m, C*H*H), 2.07–2.20 (1H, m, CH*H*), 3.28–3.34 & 3.35–3.41 (2H, m, NCH_2_), 3.76 & 3.81 (3H, 2 × s, OCH_3_), 4.12–4.17 & 4.52–4.57 (1H, m, NC*H*CHO), 5.08 & 5.12 (2H, 2 × s, OCH_2_), 6.53 & 6.61 (1H, 2 × s, ArH), 6.72 & 6.80 (1H, 2 × s, ArH), 7.24–7.39 (5H, m, ArH), 9.22 & 9.62 (1H, 2 × d, *J =* 1.4 & 1.8, CHO); δ_C_ (100 MHz, CDCl_3_): 24.9/26.4 (CH_2_), 46.8/48.6 (CH_2_), 56.3/56.4 (CH_3_), 60.4 (CH_2_), 64.8/66.5 (CH), 71.3/71.3 (CH_2_), 104.2/104.4 (CH), 111.2/111.5 (CH), 120.7/120.8 (q), 127.4 (CH), 128.3 (CH), 128.8 (CH), 135.9/136.0 (q), 147.3/147.3 (q), 150.0/150.1 (q), 167.2/167.4 (q), 198.0/199.4 (CH). IR: υ_max_ (thin film cm^−1^): 1245 (s), 1430 (s), 1454 (s), 1512 (s), 1604 (s), 1622 (s), 1731 (m), 2110 (s), 2942 (m). LRMS (ESI+): Found 381.2 [M+H]^+^, 403.1 [M+Na]^+^, 783.3 [2M+Na]^+^. HRMS (ESI+): Found 403.1375 [M+Na]^+^, C_20_H_20_N_4_O_4_ + Na^+^ requires 403.1377. 

*(1-(2'-Azidobenzenesulfonyl)pyrrolidin-2-yl)methanol* (**29**). 2-Azidobenzenesulfonic acid (2.95 g, 14.8 mmol) was heated at reflux in a 2M solution of (COCl)_2_ in dichloromethane (14.8 mL, 29.6 mmol) with a drop of DMF under an inert atmosphere of nitrogen for 5 h. The reaction was allowed to reach room temperature before the crude acid chloride was concentrated *in vacuo* and washed with dichloromethane (3 × 10 mL). K_2_CO_3_ (3.30 g, 23.7 mmol) in water (10 mL) was added in one portion to a stirring solution of L-prolinol (0.60 g, 5.9 mmol) in dichloromethane (15 mL). The crude sulfonyl chloride in dichloromethane (10 mL) was added slowly and the whole was stirred for 18 h at room temperature. The organic layer was separated and the aqueous layer was extracted with ethyl acetate (3 × 10 mL). The combined organic layers were dried (MgSO_4_), filtered, and concentrated to give the product as a pure orange oil (1.61 g, 96% from prolinol). NMR: δ_H_ (400 MHz, CDCl_3_): 1.67–1.78 (1H, m, C*H*H), 1.79–1.99 (3H, m, CH*H* + C*H*_2_), 2.79 (1H, bs, CH_2_O*H*), 3.38 (1H, dt, *J =* 6.3, 10.2, NCH*H*), 3.49–3.56 (1H, m, NC*H*H), 3.62 (1H, dd, *J =* 5.6, 11.5, C*H*HOH), 3.70 (1H, dd, *J =* 4.2, 11.5, CH*H*OH), 4.02–4.08 (1H, m, C*H*CH_2_OH), 7.27 (1H, ddd, *J =* 1.0, 7.8, 7.8, ArH), 7.32 (1H, dd, *J =* 1.0, 8.0, ArH), 7.62 (1H, ddd, *J =* 1.5, 7.8, 7.8, ArH), 8.02 (1H, dd, *J =* 1.5, 8.0, ArH); δ_C_ (100 MHz, CDCl_3_): 24.7 (CH_2_), 29.0 (CH_2_), 49.5 (CH_2_), 61.8 (CH), 65.5 (CH_2_), 119.9 (CH), 124.8 (CH), 129.0 (q), 132.6 (CH), 134.2 (CH), 138.2 (q). IR: υ_max_ (thin film cm^−1^): 819, 870, 900, 928, 992, 1043, 1069, 1122, 1146, 1155, 1199, 1264, 1287, 1323, 1439, 1471, 1574, 1583, 1660, 2120, 2876, 2953, 3172–3693 (br). LRMS (ESI+): Found 305.1 [M+Na]^+^, 587.1 [2M+Na]^+^, C_11_H_14_N_4_O_3_S + Na^+^ requires 305.1. HRMS (ESI+): Found 305.0676 [M+Na]^+^, C_11_H_14_N_4_O_3_S + Na^+^ requires 305.0679.

*(1-(2′-Azidobenzenesulfonyl)pyrrolidin-2-yl)carbaldehyde* (**31**). A 2M solution of oxalyl chloride in dichloromethane (1.88 mL, 3.75 mmol) was diluted with dichloromethane (10 mL) and cooled to −78 °C under nitrogen. DMSO (0.53 mL, 0.59 g, 7.5 mmol) in dichloromethane (10 mL) and the alcohol **29** (0.80 g, 2.83 mmol) in dichloromethane (5 mL) were each added over 10 min. The whole was maintained at −78 °C for 30 min before dropwise addition of Et_3_N (2.18 mL, 1.58 g, 15.6 mmol) after which the whole was allowed to reach room temperature. The reaction was quenched with a mixture of Et_2_O (10 mL) and H_2_O (10 mL). The organic layer was separated and the aqueous phase was extracted with dichloromethane (3 × 10 mL). The combined organic layers were dried (MgSO_4_), filtered, concentrated and purified on silica (20 g) (EtOAc/Hex; 2:3) to yield the product as a white solid (0.57 g, 72%) which rapidly decomposed to an orange oil and was used immediately in subsequent reactions. NMR: δ_H_ (400 MHz, CDCl_3_): 1.83–1.95 (2H, m, CH_2_), 1.98–2.09 (1H, m, C*H*H), 2.15–2.23 (1H, m, CH*H*), 3.40 (1H, dt, *J =* 9.7, 7.2, NC*H*H), 3.57 (1H, ddd, *J =* 5.5, 6.8, 9.7, CH*H*), 4.47 (1H, ddd, *J =* 1.9, 4.5, 8.5, C*H*CHO), 7.28 (1H, ddd, *J =* 7.8, 7.8, 1.0, ArH), 7.34 (1H, dd, *J =* 8.0, 1.0, ArH), 7.64 (1H, ddd, *J =* 7.8, 7.8, 1.6, ArH), 8.03 (1H, dd, *J =* 8.0, 1.6, ArH), 9.72 (1H, d, *J =* 1.6, CHO); δ_C_ (100 MHz, CDCl_3_): 25.0 (CH_2_), 27.7 (CH_2_), 48.8 (CH_2_), 67.1 (CH), 119.9 (CH), 124.9 (CH), 128.9 (q), 132.5 (CH), 134.4 (CH), 138.2 (q), 200.5 (CH). IR: υ_max_ (thin film cm^−1^): 820, 865, 999, 1080, 1122, 1156, 1199, 1265, 1287, 1332, 1439, 1471, 1574, 1583, 1603, 1730, 2122, 2953. HRMS (ESI+): compound degraded.

*N-(2'-Azidobenzoyl)-2-(carbethoxy-1''-ethenyl)-pyrrolidine* (**32**). The aldehyde **30** [33,34] (273 mg, 1.12 mmol) was dissolved in toluene (10 mL). (Carbethoxymethylene) triphenylphosphorane (390 mg, 1.12 mmol) was added in one portion and the whole was stirred at room temperature under an inert atmosphere of nitrogen for 12 h before being concentrated *in vacuo* and purified by silica chromatography (40 g) (EtOAc/Hex; 3:2) to yield the product **32** in the form of a yellow oil as a mixture of rotamers (178 mg, 51%). NMR: δ_H_ (400 MHz, CDCl_3_): 1.29 & 1.31 (3H, t, *J =* 7.1, 2 × COCH_2_C*H*_3_), 1.80–1.99 & 2.01–2.43 (4H, m, CH_2_), 3.21–3.28 & 3.78–3.86 (1H, m, C*H*H), 3.34–3.40 & 3.69–3.75 (1H, m, CH*H*), 4.13 & 4.21 (2H, q, *J =* 7.1 & 7.1, COC*H*_2_CH_3_), 4.24–4.30 & 4.96–5.00 (1H, m, NC*H*CH_2_), 5.46 & 6.15 (1H, dd & d, *J =* 15.6, 1.1 & 15.6, CHC*H*CO_2_Et), 6.59 & 6.94 (1H, dd & dd, *J =* 15.6, 6.4 & 15.6, 4.9, C*H*CHCO_2_Et), 7.12 (1H, dd, *J =* 7.5, 7.5, ArH), 7.14 (1H, d, *J =* 7.3, ArH), 7.19–7.25 (1H, m, ArH), 7.40 & 7.45 (2H, 2 × m, ArH); δ_C_ (100 MHz, CDCl_3_): 14.2/14.3 (CH_3_), 22.3/23.8 (CH_2_), 30.5/32.1 (CH_2_), 46.1/48.2 (CH_2_), 57.2 (CH), 60.4/60.6 (CH_2_), 118.5 (CH), 121.2/121.3 (CH), 124.9/125.2 (CH), 127.9 (CH), 129.1/129.5 (q), 130.6 (CH), 133.7/136.2 (q), 146.5 (CH), 165.8/166.5 (q), 167.1/167.4 (q). IR: υ_max_ (thin film cm^−1^): 753 (s), 1043 (m), 1093 (m), 1180 (s), 1301 (s), 1369 (m), 1414 (s), 1451 (s), 1475 (m), 1633 (s), 1716 (s), 2130 (s), 2238 (w), 2880 (m), 2979 (m). LRMS (ESI+): Found 315.1 [M+H]^+^, 337.1 [M+Na]^+^, 651.3 [2M+Na]^+^. HRMS (ESI+): Found 315.1451 [M+H]^+^, C_16_H_18_N_4_O_3_ + H^+^ requires 315.1452.

*Aziridinopyrrolobenzodiazepine* (**33**). The carbethoxy alkene **32** (178 mg, 0.567 mmol) was heated at reflux under nitrogen in CHCl_3_ (10 mL) for 48 h before being concentrated and purified by silica chromatography (20 g) (EtOAc/Hex; 3:2) to yield the product **33** as a ~1:1 mixture of two diastereoisomers in the form of a yellow oil (47 mg, 30%). NMR: δ_H_ (400 MHz, CDCl_3_) [mixture of isomers]: 1.23 (3H, t, *J =* 7.1, COCH_2_C*H*_3_, isomer “a”), 1.29 (3H, t, *J =* 7.1, COCH_2_C*H*_3_, isomer “b”), 1.74–1.80 (2H, m, CH_2_),1.81–2.27 (4H, m, 2 × CH_2_), 2.77 (1H, d, *J =* 2.6, C*H*CO_2_Et, isomer “a”), 2.87 (1H, d, *J =* 2.7, C*H*CO_2_Et, isomer “b”), 3.08 (1H, dd, *J =* 9.6, 2.6, ArNCH, isomer “a”), 3.37 (1H, dd, *J =* 8.7, 2.7, ArNCH, isomer “b”), 3.57 (1H, ddd, *J =* 7.3, 7.3, 12.0, C*H*H), 3.66–3.73 (4H, m, 2 × CH_2_), 3.74–3.79 (1H, m, CONCH), 3.78–3.87 (1H, m, CH*H*), 4.20 (2H, quartet, *J =* 7.1, COC*H*_2_CH_3_, isomer “a”), 4.23 (2H, quartet, *J =* 7.1, COC*H*_2_CH_3_, isomer “b”), 4.38 (1H, bd, *J =* 9.6, CONCH), 6.69 (1H, d, *J =* 7.4, ArH), 6.94 (1H, ddd, *J =* 7.5, 7.5, 0.9, ArH), 7.01 (1H, ddd, *J =* 7.5, 7.5, 1.0, ArH), 7.07 (1H, d, *J =* 8.0, ArH), 7.19 (1H, ddd, *J =* 7.6, 7.6, 1.6, ArH), 7.28 (1H, ddd, *J =* 7.7, 7.8, 1.6, ArH), 7.70 (2H, dd, *J =* 7.8, 1.8, ArH); δ_C_ (100 MHz, CDCl_3_): 14.2 (CH_3_), 14.5 (CH_3_), 23.1 (CH_2_), 25.6 (CH_2_), 29.5 (2 × CH_2_), 42.6 (CH), 46.4 (CH_2_), 47.0 (CH), 50.4 (CH), 56.6 (CH), 57.2 (CH), 60.1 (CH), 61.4 (CH_2_), 62.0 (CH_2_), 68.0 (CH_2_), 121.3 (CH), 122.0 (CH), 123.1 (CH), 123.3 (CH), 125.3 (q), 126.6 (q), 130.7 (CH), 131.0 (CH), 132.0 (CH), 132.2 (CH), 142.9 (q), 148.1 (q), 166.0 (q), 166.3 (q), 167.9 (q), 168.8 (q). IR: υ_max_ (thin film cm^−1^): 752 (m), 1024 (s), 1178 (s), 1217 (s), 1260 (s), 1372 (m), 1454 (m), 1503 (m), 1602 (s), 1622 (s), 1725 (s), 2871 (m), 2926 (m), 2977 (m). LRMS (ESI+): Found. 309.1 [M+Na]^+^. HRMS (ESI+): Found 309.1206 [M+Na]^+^, C_16_H_18_N_2_O_3_ + Na^+^ requires 309.1210.

*Aziridinopyrrolobenzothiadiazepine* (**35**). The aldehyde **31** (286 mg, 1.13 mmol) was dissolved in toluene (10 mL) and (carbethoxymethylene) triphenylphosphorane (500 mg, 1.43 mmol) was added in one portion and the whole was stirred at room temperature for 18 h. The reaction mixture was concentrated and purified by silica chromatography (40 g) (EtOAc/Hex; 3:2) to yield, as a single isomer, the aziridine **35** as a yellow oil (123 mg, 34%). NMR: δ_H_ (500 MHz, CDCl_3_): 1.41 (3H, t, *J =* 7.1, COCH_2_C*H*_3_), 1.75–1.80 (1H, m, C*H*H), 1.87–2.01 (1H, m, CH*H*), 2.09–2.16 (1H, m, C*H*H), 2.27–2.35 (1H, m, CH*H*), 3.19 (1H, ddd, *J =* 5.0, 9.6, 9.6, SO_2_NC*H*H), 3.67–3.73 (2H, m, SO_2_NCH*H* + CHC*H*CH), 4.08 (1H, ddd, *J =* 2.1, 7.4, 9.8, SO_2_NCH), 4.33–4.40 (2H, m, COC*H*_2_CH_3_), 4.94 (1H, d, *J =* 9.8, ArNCH), 7.43 (1H, ddd, *J =* 7.7, 7.7, 1.1, ArH), 7.55 (1H, dd, *J =* 8.0, 1.1, ArH), 7.60 (1H, ddd, *J =* 7.7, 7.7, 1.5, ArH), 8.04 (1H, dd, *J =* 8.0, 1.5, ArH); δ_C_ (125MHz, CDCl_3_): 14.1 (CH_3_), 22.7 (CH_2_), 28.9 (CH_2_), 46.4 (CH_2_), 60.7 (CH), 61.8 (CH), 63.0 (CH_2_), 85.4 (CH), 123.8 (CH), 126.6 (CH), 128.5 (CH), 131.6 (q), 133.6 (CH), 138.4 (q), 167.3 (q). IR: υ_max_ (thin film cm^−1^): 1035 (m), 1066 (m), 1092 (s), 1135 (m), 1167 (s), 1205 (m), 1247 (m), 1271 (m), 1344 (s), 1469 (s), 1503 (m), 1589 (m), 1738 (s), 2981 (m). LRMS (ESI+): Found 345.1 [M+Na]^+^, 723.2 [2M+Na]^+^. HRMS (ESI+): Found 345.0875 [M+Na]^+^, C_15_H_18_N_2_O_4_S + Na^+^ requires 345.0879.

*3-Benzyloxy-4-methoxy-11-ethyl-ethanoyl-[1,4]-pyrrolo[2,1-c]* *benzodiazepin-5-one* (**40**). The aldehyde **37** (198 mg, 0.58 mmol) was dissolved in toluene (10 mL) and (carbethoxy-methylene)triphenylphosphorane (200 mg, 0.58 mmol) was added in one portion and the whole was stirred at room temperature for 18 h. The reaction was concentrated *in vacuo* and purified by silica chromatography (30 g) (EtOAc/Hex; 4:1) to yield the product **40** as a yellow oil (52 mg, 21%). NMR: δ_H_ (500 MHz, CDCl_3_): 1.32 (3H, t, *J =* 7.2, CO_2_CH_2_C*H*_3_), 1.74–1.80 (1H, m, C*H*H), 1.93–2.19 (3H, m, CH*H* + CH_2_), 2.31–2.24 (2H, m, C*H*_2_CO_2_Et), 3.43–3.47 (1H, m, NHC*H*), 3.62–3.66 (1H, m, NHC*H*CH), 3.68–3.73 (1H, m, NC*H*H), 3.76–3.80 (1H, m, NCH*H*), 3.89 (3H, s, OMe), 4.22 (2H, q, *J =* 7.2, CO_2_C*H*_2_CH_3_), 5.13 (1H, d, *J =* 12.3, PhC*H*HO), 5.18 (1H, d, *J =* 12.3, PhCH*H*O), 6.34 (1H, s, ArH), 7.31–7.50 (7H, m, 6 × ArH + NH); δ_C_ (125 MHz, CDCl_3_): 14.2 (CH_3_), 23.2 (CH_2_), 29.9 (CH_2_), 37.3 (CH_2_), 46.9 (CH_2_), 56.3 (CH_3_), 60.1 (CH), 61.0 (CH_2_), 62.7 (CH), 70.9 (CH_2_), 108.1 (CH), 113.1 (CH), 120.0 (q), 127.4 (CH), 128.0 (CH), 128.4 (CH), 136.5 (q), 137.8 (q), 145.0 (q), 150.9 (q), 168.3 (q), 171.9 (q). IR: υ_max_ (thin film cm^−1^): 723 (m), 1025 (s), 1119 (s), 1178 (s), 1218 (s), 1260 (s), 1373 (m), 1432 (s), 1453 (m), 1503 (m), 1602 (s), 1623 (m), 1726 (m), 2860 (m), 2924 (s), 2953 (m). LRMS (ESI+): Found 447.2 [M+Na]^+^. HRMS (ESI+): Found 447.1895 [M+Na]^+^ requires C_24_H_28_N_2_O_5_ + Na^+^ requires 447.1890.

### 3.3. Synthesis and Reactivity of the Azetidino-Based Systems

*4-Methyl-4-ethenyl-1-azetidin-2-one* (**41a**). To a stirred solution of isoprene (2.33 g, 3.43 mL, 34.41 mmol) in dry diethyl ether (15 mL) at –78 °C was added a solution of chlorosulfonyl isocyanate (4.88 g, 3.01 mL, 34.01 mmol) in dry ether (10 mL) dropwise over one hour. The reaction mixture was allowed to warm to −10) dropwise over one hour. The reaction mixture was allowed to warm to −10 °C and then the reaction flask was transferred to an ice-salt bath and stirred for 30 min. The cooled solution was added dropwise to a vigorously stirred solution of water (50 mL), sodium carbonate (9.00 g), sodium sulfite (6.01 g) and ice (30 g) over 10 min. The mixture was stirred at −10 °C for 1 h and then allowed to warm to room temperature and extracted with diethyl ether (6 × 20 mL). The combined organic extracts were dried (MgSO_4_) and the solvent removed under reduced pressure to give the product as a pale yellow oil (2.20 g, 58%). NMR: δ_H_ (400 MHz, CDCl_3_): 1.50 (3H, s, Me), 2.79 (2H, s, C*H*_2_), 5.10 (1H, dd, *J =* 10.6, 0.7, CH=C*H*H), 5.22 (1H, dd, *J =* 17.2, 0.7, CH=CH*H*), 6.02 (1H, dd, *J =* 10.6, 17.2, C*H*=CH_2_), 6.83 (1H, br, NH); δ_C_ (100 MHz, CDCl_3_): 24.8 (CH_2_), 50.7 (CH_3_), 54.5 (q), 113.8 (CH), 141.1 (CH_2_), 167.6 (q). IR: υ_max_ (thin film cm^−1^): 923 (m), 1153 (m), 1186 (m), 1226 (m), 1274 (w), 1304 (m), 1372 (m), 1412 (m), 1643 (m), 1720 (s), 2970 (w), 3235 (m). LRMS (ESI+): Found 134.1 [M+Na]^+^, 291.2 [2M+3Na]^+^.

*4-Methyl-4-ethenyl-1-azetidin-2-thione* (**43a**). To the azetidin-2-one (0.55 g, 4.97 mmol) in dry THF (15 mL) was added Lawesson’s reagent (1.00 g, 2.48 mmol) and the whole was stirred at room temperature for an hour before being heated to reflux for two h. The reaction was cooled to ambient temperature before being concentrated under reduced pressure and purified by silica chromatography (75 g) (EtOAc/petroleum ether; 1:3) to give the product as a yellow oil (0.35 g, 56%). The reaction was higher yielding (up to 70%) on a larger scale (2 g of lactam), but less convenient to purify (stench). NMR: δ_H_ (400 MHz, CDCl_3_): 1.60 (3H, s, Me), 2.98 (2H, s, CSCH_2_), 5.21 (1H, d, *J =* 10.6, CH=C*H*H), 5.28 (1H, d, *J =* 17.2, CH=CH*H*), 6.05 (1H, dd, *J =* 10.6, 17.2 C*H*=CH_2_), 8.78 (1H, bs, NH); δ_C_ (100 MHz, CDCl_3_): 23.8 (CH_3_), 54.6 (CH_2_), 63.7 (q), 115.1 (CH_2_), 139.0 (CH), 202.2 (q). IR: υ_max_ (thin film cm^−1^): 924 (s), 989 (m), 1016 (m), 1081 (s), 1217 (m), 1288 (m), 1374 (m), 1404 (s), 1466 (s), 2971 (w), 3147 (m).

*4-Ethenyl-2-azetidinone* (**41b**). To 1,3-butadiene (10 mL) condensed into anhydrous ether (40 mL) at −10 °C, was added a solution of chlorosulfonyl isocyanate (3.0 mL, 34.5 mmol) in anhydrous ether (10 mL) over one hour, under an atmosphere of nitrogen. The temperature was maintained at −10 °C for a further period of 3 h and warmed slowly to room temperature overnight, to produce a clear, yellow solution. The solution was added to an ice cold mixture of water (70 mL), ice (30 g), NaHCO_3_ (9.0 g) and Na_2_SO_3_ (6.0 g) and stirred for one hour at −10 °C. The reaction was allowed to warm to room temperature before being extracted with ether (6 × 20 mL). The combined organic layers were dried over anhydrous sodium sulfate, filtered and evaporated to dryness under reduced pressure to yield 4-ethenyl-2-azetidinone (2.01 g, 60% yield) as a clear yellow oil. NMR: δ_H_ (400 MHz, CDCl_3_): 6.49 (1H, s, N−H), 5.92 (1H, ddd, *J =* 17.2, 10.2, 3.2, =CH), 5.32 (1H, d, *J =* 17.2, =CH_2_), 5.20 (1H, d, *J =* 10.2, =CH_2_), 4.13 (1H, m, NCH), 3.25 (1H, m, ring-CH_2_), 2.70 (1H, d, *J =* 17.2 ring-CH_2_); δ_C_ (100 MHz, CDCl_3_): 167.82 (C=O), 137.43 (CH), 116.93 (CH_2_), 49.40 (CH_2_), 48.93 (CH). IR: υ_max_ (thin film cm^−1^): 3274.6 (m, broad), 1755.4 (s), 1448.2 (m), 1413.8 (m), 1380.1 (m), 1254.7 (w).

*4-Ethenylazetidin-2-thione* (**43b**). To the lactam (1.00 g, 10.3 mmol) dissolved in anhydrous THF (15 mL), was added Lawesson’s reagent (2.08 g, 5.15 mmol) in one portion, with stirring. The reaction was stirred at ambient temperature, under an inert atmosphere of dry nitrogen for one hour, before being heated at reflux for an additional hour. The reaction was monitored for completion by tlc and was subsequently cooled to room temperature, to give a crude product as a clear orange liquid. The sample was concentrated by rotary evaporation and purified using silica column chromatography (eluent: petroleum ether-ethyl acetate 4:1), to yield 4-ethenyl-azetidin-2-thione (0.67 g, 58%) as a clear, yellow oil. NMR: δ_H_ (400 MHz, CDCl_3_): 8.68 (1H, s, N−H), 5.96 (1H, ddd, *J =* 17.1, 10.3, 3.0, =CH), 5.35 (1H, d, *J =* 17.1, =CH_2_), 5.26 (1H, d, *J =* 10.3, =CH_2_), 4.62 (1H, m, NCH), 3.30 (1H, m, ring-CH_2_), 2.84 (1H, m, ring-CH_2_); δ_C_ (100 MHz, CDCl_3_): 203.65 (C=S), 135.14 (CH), 118.34 (CH_2_), 58.01 (CH), 48.34 (CH_2_). IR: υ_max_ (thin film cm^−1^): 3174.1 (m, broad), 1482.6 (s), 1426.0 (m), 1407.2 (w), 1237.1 (m). 

*1-(2′-Azidobenzoyl)-4-methyl-4-vinylazetidin-2-one* (**42a**). 2-Azidobenzoic acid (508 mg, 3.02 mmol) was heated to reflux in SOCl_2_ (4 mL) for 3 h under nitrogen. The excess SOCl_2_ was removed *in vacuo* and the crude acid chloride was dissolved in DCM (3 × 5 mL) which was removed *in vacuo* to yield the crude 2-azidobenzoylchloride. The β-lactam **41** (457 mg, 4.12 mmol) in DCM (25 mL) and DMAP (100 mg) were chilled to −10 °C. The crude acid chloride in DCM (5 mL) was added dropwise to the solution over 10 min. The whole was maintained at −10 °C for 30 min before the addition of Et_3_N (0.89 mL, 646 mg, 6.40 mmol) and the whole was allowed to reach room temperature overnight. The reaction was concentrated under reduced pressure and purified by silica chromatography (70 g) (EtOAc/Hex; 1:4) to give the product **42a** as a mixture of rotamers in the form of a dark yellow oil (498 mg, 65%). NMR: δ_H_ (400 MHz, CDCl_3_) *rotamer 1*: 1.87 (3H, s, Me), 2.98 (1H, d, *J =* 16.2, COC*H*H), 3.08 (1H, d, *J =* 16.2, COCH*H*), 5.35 (1H, d, *J =* 10.7, C*H*H=CH), 5.45 (1H, d, *J =* 17.3, CH*H*=CH), 6.24 (1H, dd, *J =* 10.7, 17.3, C*H*=CH_2_), 7.22 (1H, dd, *J =* 7.6, 7.6, ArH), 7.23 (1H, d, *J =* 8.3, ArH), 7.42 (1H, dd, *J =* 7.6, 1.4, ArH), 7.52 (1H, ddd, *J =* 8.3, 8.3, 1.4, ArH). δ_H_ (400 MHz, CDCl_3_) *rotamer 2*: 1.51 (3H, s, Me), 2.68 (1H, d, *J =* 15.8, COC*H*H), 2.82 (1H, d, *J =* 15.8, COCH*H*), 5.20 (1H, d, *J =* 10.6, C*H*HCH), 5.32 (1H, d, *J =* 17.2, CH*H*CH), 5.91 (1H, dd, *J =* 10.6, 17.2, C*H*CH_2_), 7.21 (1H, ddd, *J =* 7.6, 7.6, 0.9, ArH), 7.24 (1H, d, *J =* 7.6, ArH), 7.51 (1H, ddd, *J =* 7.8, 7.8, 1.5, ArH), 7.67 (1H, dd, *J =* 7.8, 1.5, ArH); δ_C_ (100 MHz, CDCl_3_) *rotamer 1*: 21.7 (CH_3_), 49.2 (CH_2_), 58.9 (q), 114.9 (CH_2_), 117.5 (CH), 123.7 (CH), 125.8 (q), 128.1 (CH), 131.2 (CH), 137.1 (q), 137.2 (CH), 162.4 (q), 162.5 (q); δ_C_ (100 MHz, CDCl_3_) *rotamer 2*: 27.6 (CH_3_), 39.5 (CH_2_), 58.6 (q), 114.3 (CH_2_), 119.9 (CH), 123.5 (q), 124.7 (CH), 130.8 (CH), 132.1 (CH), 138.9 (q), 139.9 (CH), 151.5 (q), 165.3 (q). IR: υ_max_ (thin film cm^−1^): 1084 (m), 1216 (s), 1329 (s), 1391 (s), 1451 (m), 1478 (m), 1519 (s), 1604 (m), 1656 (m), 1682 (m), 1801 (s), 2131 (s), 2853 (m), 2925 (m). LRMS (ESI+): 279.1 [M+Na]^+^, 535.2 [2M+Na]^+^. C_13_H_12_N_4_NaO_2_ requires 279.1. HRMS (ESI+): 279.0862 [M+Na]^+^, C_13_H_12_N_4_NaO_2_ requires 279.0852. 

*8,9-Dimethylazetidino[2,1-a][1,4]benzodiazepin-2,11-dione* (**45**). The azetidinone **42a** (250 mg, 0.97 mmol) was heated to reflux in CHCl_3_ (10 mL) under an atmosphere of dry nitrogen and monitored by NMR every 24 h. After 72 h, the reaction mixture was concentrated under reduced pressure and purified by silica chromatography (22 g) using graduated elution (EtOAc/Hex; 1:4–3:1) to give the imine **45** as a yellow oil (84 mg, 38%). NMR: δ_H_ (500 MHz, CDCl_3_): 2.00 (3H, s, Me), 2.49 (1H, s, Me), 3.42 (1H, d, *J =* 16.0, COCH*H*), 3.77 (1H, d, *J =* 16.0, COCH*H*), 7.52 (1H, ddd, *J =* 7.7, 7.7, 1.0, Ar*H*), 7.70 (1H, dd, *J =* 8.0, 1.0, Ar*H*), 7.78 (1H, ddd, *J =* 7.7, 7.7, 1.6, Ar*H*), 8.32 (1H, dd, *J =* 8.0, 1.6, Ar*H*); δ_C_ (125MHz, CDCl_3_): 20.1 (CH_3_), 26.2 (CH_3_), 43.6 (CH_2_), 73.3 (q), 123.7 (q), 126.5 (CH), 126.7 (CH), 127.4 (CH), 134.3 (CH), 149.7 (q), 155.2 (q), 158.1(q), 204.2 (q). IR: υ_max_ (thin film cm^−1^): 702 (s), 730 (s), 1264 (s), 1421 (m), 1463 (m), 1609 (m), 1657 (m), 1686 (m), 1721 (s), 1799 (m), 2928 (w). LRMS (ESI+): Found 229.1 [M+H]^+^, 251.1 [M+Na]^+^, 479.2 [2M+Na]^+^. HRMS (ESI+): Found 229.0978 [M+H]^+^, 479.1703 [2M+Na], C_13_H_13_N_2_O_2_ requires 229.0972 and C_26_H_24_N_4_NaO_4_ requires 479.1690.

*1-(2*′*-Azidobenzoyl)-4-methyl-4-vinyl-1-azetidin-2-thione* (**44a**). 2-Azidobenzoic acid (214 mg, 1.3 mmol) was heated to reflux under nitrogen in SOCl_2_ (4 mL) for 4 h. The excess thionyl chloride was removed *in vacuo* and the product was dissolved in DCM (3 × 5 mL) and concentrated *in vacuo* to give the crude acid chloride. The thiolactam (250 mg, 1.97 mmol) in DCM (25 mL) and DMAP (100 mg) was chilled to −10 °C. The crude acid chloride in DCM (5 mL) was added dropwise over 10 min and the whole was maintained at −10 °C for 30 min before dropwise addition of Et_3_N (0.41 mL, 299 mg, 2.95 mmol). The whole was allowed to reach room temperature overnight and the reaction was concentrated and purified by silica chromatography (22 g) (EtOAc/Hex; 1:4) to give the product **44a** as an orange oil (296 mg, 84%). NMR: δ_H_ (400 MHz, CDCl_3_): 1.92 (3H, s, Me), 2.93 (1H, d, *J =* 16.9, CSC*H*H), 3.04 (1H, d, *J =* 16.9, CSCH*H*), 5.38 (1H, d, *J =* 10.8, C*H*H=CH), 5.45 (1H, d, *J =* 17.3, CH*H=*CH), 6.31 (1H, dd, *J =* 17.3, 10.8 CHH=C*H*), 7.20 (1H, dd, *J =* 8.2, 0.8, ArH), 7.24 (1H, ddd, *J =* 7.6, 7.6, 0.8, ArH), 7.36 (1H, dd, *J =* 7.6, 1.5, ArH), 7.54 (1H, ddd, *J =* 8.2, 8.2, 1.5, ArH); δ_C_ (100 MHz, CDCl_3_): 22.3 (CH_3_), 54.5 (CH_2_), 67.5 (q), 116.4 (CH_2_), 118.5 (CH), 125.1 (CH), 126.6 (q), 128.9 (CH), 132.1 (CH), 137.7 (CH), 138.3 (q), 164.2 (q), 201.7 (q). IR: υ_max_ (thin film cm^−1^): 909 (s), 931 (m), 1215 (s), 1322 (s), 1358 (m), 1448 (w), 1478 (m), 1674 (s), 2131 (s). LRMS (ESI+): Found 295.1 [M+Na]^+^, 567.1 [2M+Na]^+^. HRMS (ESI+): Found 295.0623 [M+Na]^+^, C_13_H_12_N_4_NaOS requires 295.0624. 

*12-Methyl-1,2,3-triazolino[1,5-a]azetidino[1,4-c][1,4]benzodiazepin-2-on-14-thione* (**46**). The 1-(2'-azidobenzoyl)-azetidin-2-thione **44a** (295 mg, 1.08 mmol) was heated at reflux in CHCl_3_ (10 mL) under nitrogen for 36 h before being concentrated under reduced pressure and purified by silica chromatography (22 g) (EtOAc/Hex; 1:1) to give the triazolino product **46** as a yellow oil (180 mg, 61%). NMR: δ_H_ (500 MHz, CDCl_3_): 1.22 (3H, s, Me), 2.89 (1H, d, *J =* 16.8, SCC*H*H), 2.94 (1H, d, *J =* 16.8, SCCH*H*), 4.27 (1H, dd, *J =* 6.1, 12.2, CH_3_C*H*N), 4.37 (1H, dd, *J =* 6.1, 17.7, N_3_C*H*H), 4.71 (1H, dd, *J =* 12.2, 17.7, N_3_CH*H*), 7.14 (1H, ddd, *J =* 7.1, 7.1, 1.0, ArH), 7.52 (1H, ddd, *J =* 7.2, 7.2, 1.6, ArH), 8.04 (1H, dd, *J =* 8.4, 1.0, ArH), 8.2 (1H, dd, *J =* 8.4, 1.6, ArH); δ_C_ (125 MHz, CDCl_3_): 16.8 (CH_3_), 50.5 (CH_2_), 59.6 (CH), 65.0 (q), 70.4 (CH_2_), 115.9 (q), 118.6 (CH), 123.4 (CH), 134.2 (CH), 134.5 (CH), 138.3 (q), 161.8 (q), 198.1 (q). IR: υ_max_ (thin film cm^−1^): 745 (s), 1103 (m), 1162 (m), 1211 (m), 1251 (m), 1322 (s), 1461 (s), 1483 (s), 1607 (s), 1651 (s), 1678 (s), 1720 (m), 2921 (w), 3000 (w). LRMS (ESI+): Found 295.1 [M+Na]^+^, 567.1 [2M+Na]^+^. HRMS (ESI+): Found 295.0622 [M+Na]^+^, C_13_H_12_N_4_NaOS requires 295.0624. 

*11-Thioxo-8,9-dimethyl-azetidino[2,1-c][1,4]benzodiazepin-2-one* (**47**). A sample of the triazolo compound **46** from above (87 mg, 0.32 mmol) was heated at reflux in CHCl_3_ (10 mL) under nitrogen for a week before being concentrated under reduced pressure and purified by silica chromatography (15 g) (EtOAc/Hex; 3:2) to give the methyl imine product as a yellow oil (25 mg, 32%). NMR: δ_H_ (400 MHz, CDCl_3_): 1.98 (3H, s, Me), 2.47 (3H, s, Me), 3.40 (1H, d, *J =* 16.0, CSC*H*H), 3.77 (1H, d, *J =* 16.0, CSCH*H*), 7.50 (1H ddd, *J =* 7.7, 7.7, 1.0, ArH), 7.68 (1H, d, *J =* 8.1, ArH), 7.77 (1H, ddd, *J =* 7.7, 7.7, 1.4, ArH), 8.30 (1H, dd, *J =* 8.1, 1.4, ArH); δ_C_ (100 MHz, CDCl_3_): 20.1 (CH_3_), 26.3 (CH_3_), 43.6 (CH_2_), 73.3 (q), 123.6 (q), 126.6 (CH), 126.7 (CH), 127.3 (CH), 134.3 (CH), 149.6 (q), 155.2 (q), 158.0 (q), 204.4 (q). IR: υ_max_ (thin film cm^−1^): 647 (s), 770 (s), 1103 (m), 1115 (m), 1132 (m), 1300 (m), 1323 (s), 1346 (s), 1460 (s), 1606 (s), 1651 (s), 1676 (s), 2928 (w), 2975 (w). LRMS (ESI+): Found 267.1 [M+Na]^+^. HRMS (ESI+): Found 267.0551 [M+Na]^+^, C_13_H_12_N_2_NaOS requires 267.0563.

*1-(*2′*-Azidobenzoyl)-4-ethenyl-1-azetidin-2-thione* (**44b**). To a solution of 4-ethenyl-1-azetidin-2-thione (**43b**, 0.16 g, 1.42 mmol) and dimethylaminopyridine (0.1 g, 0.82 mmol) in anhydrous dichloromethane (20 mL), was added, with stirring and under an atmosphere of dry nitrogen, 2-azidobenzoylchloride (0.28 g, 1.56 mmol) in anhydrous dichloromethane (10 mL) [prepared as described previously], dropwise over 20 min at −10 °C. The reaction was stirred for 30 min before triethylamine (0.29 mL, 2.83 mmol) was added dropwise over 10 min at −10 °C. The reaction mixture was warmed to room temperature and left to stir at ambient temperature for 24 h, before being concentrated by rotary evaporation under reduced pressure and purified by flash silica column chromatography (eluent: petroleum ether-ethyl acetate, 4:1) to yield 1-(*o*-azidobenzoyl)-4-ethenyl-1-azetidin-2-thione (0.26 g, 71%) as a yellow solid, melting point: 79–82 °C. NMR: δ_H_ (500 MHz, CDCl_3_): 2.84 (1H, dd, *J =* 17.1, 3.1, CH*H*). 3.24 (1H, dd, *J =* 17.1, 5.9, C*H*H), 5.15 (1H, m, CH), 5.38 (1H, d, *J =* 10.4, CH*H*=CH), 5.49 (1H, d, *J =* 17.2, C*H*H=CH), 6.09 (1H, ddd, *J =* 17.2, 10.4, 7.0, =CH), 7.21 (2H, m, 2 × ArH), 7.39 (1H, dd, *J =* 7.6, 1.2, ArH), 7.53 (1H, ddd, *J =* 7.8, 7.8, 1.5, ArH); δ_C_ (125 MHz, CDCl_3_): 47.10 (CH_2_), 59.42 (CH), 118.52 (CH), 119.46 (CH_2_), 124.97 (CH), 126.06 (q), 129.32 (CH), 132.40 (CH), 133.59 (CH), 163.97 (C=O), 201.89 (C=S). IR: υ_max_ (thin film cm^−1^): 2927.9 (s), 2853.4 (s), 2128.3 (s), 1687.0 (s), 1462.9 (m), 1375.5 (m), 1303.6 (m). HRMS (ESI+): calc. for C_12_H_10_N_4_OS + H^+^ = 259.0648, measured = 259.0648. 

*(Aziridino[1,2-a]azetidino[2,1-c])[1,4]benzodiazepine-7-one-9-thione* (**48**). A solution of 1-(2'*-*azidobenzoyl)-4-ethenyl-1-azetidin-2-thione (**44b**, 0.074 g, 2.87 mmol) dissolved in anhydrous toluene (6 mL) was heated at reflux under an atmosphere of dry nitrogen for 24 h at which point TLC confirmed that the reaction had gone to completion. The sample was cooled to room temperature, concentrated under reduced pressure, and purified by silica column chromatography (eluent: petroleum ether:ethyl acetate, 1:2) to yield the title compound **48** (0.032 g, 48% yield) as a yellow oil. NMR: δ_H_ (500 MHz, CDCl_3_): 2.26 (1H, d, *J =* 3.5, NCH*H*), 2.81 (1H, d, *J =* 4.4, NC*H*H), 3.10 (1H, dd, *J =* 16.8, 2.8, CSCH*H*), 3.17 (1H, ddd, *J =* 8.9, 4.4, 3.5, ArNC*H*), 3.31 (1H, dd, *J =* 16.8, 5.7, CSC*H*H), 4.20 (1H, ddd, *J =* 8.9, 5.7, 2.8, CONC*H*), 7.10 (1H, ddd, *J =* 7.6, 7.6, 0.8, ArH), 7.17 (1H, d, *J =* 8.1, ArH), 7.45 (1H, ddd, *J =* 7.7, 7.7, 1.6, ArH), 7.80 (1H, dd, *J =* 8.1, 1.6, ArH); δ_C_ (125 MHz, CDCl_3_): 35.13 (CH_2_), 42.00 (CH), 45.14 (CH_2_), 58.86 (CH), 122.90 (CH), 123.47 (CH), 132.20 (CH), 133.76 (CH), 150.04 (q), 163.89 (C=O), 199.55 (C=S). IR: υ_max_ (thin film cm^−1^): 2873.3 (w), 1698.2 (s), 1653.5 (s), 1601.9 (m), 1483.4 (m), 1355.8 (m), 1345.6 (s), 1195.4 (m). HRMS (ESI+): calc. for C_12_H_10_N_2_OS + H^+^ = 231.0587, measured = 231.0588.

## 4. Conclusions

Intramolecular 1,3-dipolar cycloadditions between an azide and an alkene in 1-(2'-azidoaroyl)-2-alkenyl-proline based systems led to aziridino-fused pyrrolobenzodiazepines and pyrrolobenzothiadiazepines (4 examples), or to pyrrolobenzodiazepines with a methyl substituted imine (1 example) or a pyrrolobenzodiazepine with a carbethoxymethylene substituted amine (1 example). 1-(2'-Azidoaroyl)-4-alkenyl-azetidin-2-ones reacted to give the corresponding aziridino-fused azetidinobenzodiazepine (1 example), triazolino-fused azetidinobenzodiazepine (1 example) or azetidinobenzodiazepine with a methyl substituted imine (2 examples). These reactivity patterns are unpredictable but can all be rationalized by the formation of a common triazoline intermediate. We are currently further exploring the chemistry of the 4-ethenyl-1-azetidin-2-one derivatives **41**–**44** and are also investigating the antitumour and antibiotic potential of the PBD, PBTD and azetidinobenzodiazepines reported in this paper.

## References

[1] Hochlowski J.E., Andres W.W., Theriault R.J., Jackson M., McAlpine J.B. (1987). Abbeymycin, a new anthramycin-type antibiotic produced by a streptomycete. J. Antibiot..

[2] Wang T., Lui A.S., Cloudsdale I.S. (1999). A novel route to pyrrolo[2,1-c][1,4]benzodiazepin-5-ones. Formal total synthesis of (±)-DC-81. Org. Lett..

[3] Pettersson B., Hasimbegovic V., Bergman J. (2011). One-pot Eschenmoser episulfide contractions in DMSO: Applications to the synthesis of fuligocandins A and B and a number of vinylogous amides. J. Org. Chem..

[4] Gerratana B. (2012). Biosynthesis, synthesis and biological applications of pyrrolobenzodiazepines. Med. Res. Rev..

[5] Antonow D., Thurston D.E. (2011). Synthesis of DNA-interactive pyrrolo[2,1-c][1,4]benzodiazepines (PBDs). Chem. Rev..

[6] Brucoli F., Hawkins R.M., James C.H., Jackson P.J.M., Wells G., Jenkins T.C., Ellis T., Kotecha M., Hochhauser D., Hartley J.A. (2013). An extended pyrrolobenzodiazepine-polyamide conjugate with selectivity for a DNA sequence containing the ICB2 transcription factor binding site. J. Med. Chem..

[7] Kamal A., Ramakrishna G., Janaki Ramaiah M., Viswanath A., Subba Rao A.V., Bagul C., Mukhopadyay D., Pushpavalli S.N.C.V.L., Pal-Bhadra M. (2013). Design, synthesis and biological evaluation of imidazo[1,5-a]pyridine–PBD conjugates as potential DNA-directed alkylating agents. Med. Chem. Commun..

[8] Rahman K.M., Jackson P.J.M., James C.H., Piku Basu B., Hartley J.A., de la Fuente M., Schatzlein A., Robson M., Barbara Pedley R., Pepper C. (2013). GC-targeted C8-linked pyrrolobenzodiazepine-biaryl conjugates with femtomolar *in vitro* cytotoxicity and *in vivo* antitumor activity in mouse models. J. Med. Chem..

[9] Hartley J.A., Hamaguchi A., Suggitt M., Gregson S.J., Thurston D.E., Howard P.W. (2012). DNA interstrand cross-linking and *in vivo* antitumor activity of the extended pyrrolo[2,1-c][1,4]benzodiazepine dimer SG2057. Investig. New Drugs.

[10] Gregson S.J., Howard P.W., Hartley J.A., Brooks N.A., Adams L.J., Jenkins T.C., Kelland L.R., Thurston D.E. (2001). Design, synthesis, and evaluation of a novel pyrrolobenzodiazepine DNA-interactive agent with highly efficient cross-linking ability and potent cytotoxicity. J. Med. Chem..

[11] Rahman K.M., James C.H., Thurston D.E. (2011). Effect of base sequence on the DNA cross-linking properties of pyrrolobenzodiazepine (PBD) dimers. Nucleic Acids Res..

[12] Witt A., Bergman J. (2001). Total syntheses of the benzodiazepine alkaloids curcumdatin F and curcumdatin C. J. Org. Chem..

[13] López-Romero B., Evrard G., Durant F., Sevrin M., George P. (1998). Molecular structure and stereoelectronic properties of sarmazenil—A weak inverse agonist at the omega modulatory sites (benzodiazepine receptors): Comparison to bretazenil and flumazenil. Bioorg. Med. Chem..

[14] Mohapatra D.K., Maity P.K., Shabab M., Khan M.I. (2009). Click chemistry based rapid one-pot synthesis and evaluation for protease inhibition of new tetracyclic fused benzodiazepine derivatives. Bioorg. Med. Chem. Lett..

[15] Thurston D.E., Bose D.S. (1994). Synthesis of DNA-interactive pyrrolo[2.1-c][1,4]benzodiazepines. Chem. Rev..

[16] Markandeya N., Shankaraiah N., Reddy C.S., Santos L.S., Kamal A. (2010). Asymmetric syntheses of piperidino-benzodiazepines through ‘cation-pool’ host/guest supramolecular approach and their DNA-binding studies. Tetrahedron Asymmetry.

[17] Thurston D.E., Jones G.B., Davis M.E. (1990). Synthesis and reactivity of a novel oxazolo[2,3-c][1,4]benzodiazepine ring system with DNA recognition potential: A new class of anthramycins. J. Chem. Soc. Chem. Commun..

[18] Tozuka Z., Yazawa H., Murata M., Takaya T. (1983). Studies on tomaymycin. III. Synthesis and antitumor activity of tomaymycin analogs. J. Antibiot..

[19] Stefancich G., Artico M., Massa S., Corelli F. (1981). Research on nitrogen heterocyclic compounds; XIII. Synthesis of 5H-imidazo[2,1-c][1,4]benzodiazepine, a novel tricyclic ring system. Synthesis.

[20] Mitra S., Darira H., Chattopadhyay P. (2013). Efficient synthesis of imidazole-fused benzodiazepines using palladium-catalyzed intramolecular C−N bond formation reaction. Synthesis.

[21] Basolo L., Beccalli E.M., Borsini E., Broggini G., Khansaa M., Rigamonti M. (2010). Access to a novel class of tetracyclic 1,4-benzodiazepin-5-ones starting from α-amino acids by Pd-catalysed amination/1,3-dipolar cycloaddition as the key steps. Eur. J. Org. Chem..

[22] Hemming K., Loukou C. (2005). Synthetic approaches to 1,2,5-benzothiadiazepine 1,1-dioxides—Sulfonamide analogues of the 1,4-benzodiazepines. J. Chem. Res..

[23] Artico M., Silvestri R., Pagnozzi E., Stefancich G., Massa S., Loi A.G., Putzolu M., Corrias S., Spiga M.G., La Colla P. (1996). 5*H*-Pyrrolo[1,2-b][1,2,5]benzothiadiazepines (PBTDs): A novel class of non-nucleoside reverse transcriptase inhibitors. Bioorg. Med. Chem..

[24] Di Stefano C., Marfe G., Trawinska M.M., Sinibaldi-Salimei P., Silvestri R., Amadori S., Abruzzese E. (2010). Pyrrolo[1,2-b][1,2,5]benzothiadiazepines (PBTDs) exert their anti-proliferative activity by interfering with Akt-mTOR signaling and bax: Bcl-2 ratio modulation in cells from chronic myeloid leukemic patients. Cancer Sci..

[25] Hemming K., Chambers C.S., Hamasharif M.S., João H., Khan M.N., Patel N., Airley R., Day S. (2014). Azide based routes to tetrazolo and oxadiazolo derivatives of pyrrolobenzodiazepines and pyrrolobenzothiadiazepines. Tetrahedron.

[26] Chambers C.S., Patel N., Hemming K. (2010). Intramolecular 1,3-dipolar cycloaddition as a route to triazolobenzodiazepines and pyrrolobenzodiazepines. Tetrahedron Lett..

[27] Patel N., Chambers C.S., Hemming K. (2009). Synthesis of benzothiadiazines, benzothiadiazepines, and benzothiadiazocines from intramolecular azide reactions and iodocyclizations. Synlett.

[28] Broggini G., Garanti L., Molteni G., Pilati T. (2001). Stereoselective intramolecular cycloadditions of homochiral *N*-alkenoyl aryl azides. Tetrahedron Asymmetry.

[29] Beccalli E., Broggini G., Paladino G., Pilati T., Pontremoli G. (2004). Diastereoselective synthesis of enantiopure (α*R*)-2-methyl-4-(α-phenylethyl)-1,2,3,4-tetrahydro-benzo[*e*][1,4]diazepine-5-ones. Tetrahedron Asymmetry.

[30] Bass P.D., Gubler D.A., Judd T.C., Williams R.M. (2013). Mitomycinoid alkaloids: Mechanism of action, biosynthesis, total syntheses, and synthetic approaches. Chem. Rev..

[31] Sato T., Tsujimoto K., Matsubayashi K.-I., Ishibashi H., Ikeda M. (1992). Carbamoylmethyl radical cyclization: Formal synthesis of (−)-trachelanthamidine. Chem. Pharm. Bull..

[32] Broggini G., de Marchi I., Paladino G., Pilati T., Terraneo A. (2005). Effective synthesis of enantiopure [1,2,3]triazolo[1,5-a]- and pyrazolo[1,5-a]-pyrrolo[2,1-c][1,4]benzodiazepines by diastereoselective intramolecular azide and nitrilimine cycloadditions. Synthesis.

[33] Molina P., Díaz I., Tárraga A. (1995). Synthesis of pyrrolo[2,1-c][1,4]benzodiazepines via intramolecular aza-Wittig reaction. Synthesis of the antibiotic DC-81. Tetrahedron.

[34] Eguchi S., Yamashita K., Matsushita Y., Kakehi A. (1995). Facile synthesis of 1,4-benzodiazepin-5-one derivatives via intramolecular aza-Wittig reaction. Application to an efficient synthesis of *O*-benzyl DC-81. J. Org. Chem..

[35] Tsang W.Y., Ahmed N., Hemming K., Page M.I. (2008). An activated sulfonylating agent that undergoes general base-catalysed hydrolysis by amines in preference to aminolysis. J. Org. Chem..

[36] Hemming K., O’Gorman P.A., Page M.I. (2006). The synthesis of azabicyclo[4.2.1]nonenes by the addition of a cyclopropenone to 4-vinyl substituted 1-azetines—Isomers of the homotropane nucleus. Tetrahedron Lett..

[37] Blackburn C., Achab A., Elder A., Ghosh S., Guo J., Harriman G., Jones M. (2005). Synthesis of 3-amino-1,2,4-benzothiadiazine 1,1-dioxides via a tandem aza-Wittig/heterocumulene annulation. J. Org. Chem..

